# Induction Chemotherapy Followed by Primary Tumor Resection Did Not Bring Survival Benefits in Colon Cancer Patients With Asymptomatic Primary Lesion and Synchronous Unresectable Metastases

**DOI:** 10.3389/fonc.2022.747124

**Published:** 2022-01-31

**Authors:** Mingzhu Huang, Ya’nan Yang, Qingguo Li, Chenchen Wang, Lei Liang, Xiaodong Zhu, Wen Zhang, Zhiyu Chen, Dan Huang, Wenhua Li, Xiaowei Zhang, Xiaoying Zhao, Lixin Qiu, Qirong Geng, Nuoya Yu, Wenfang Du, Sijie Sun, Xuedan Sheng, Xinxiang Li, Weijian Guo

**Affiliations:** ^1^ Department of Gastrointestinal Medical Oncology, Fudan University Shanghai Cancer Center, Shanghai, China; ^2^ Department of Oncology, Shanghai Medical College, Fudan University, Shanghai, China; ^3^ Department of Colorectal Surgery, Fudan University Shanghai Cancer Center, Shanghai, China; ^4^ Department of Pathology, Fudan University Shanghai Cancer Center, Shanghai, China

**Keywords:** colorectal cancer, primary tumor resection, unresectable metastases, chemotherapy, prognosis

## Abstract

**Background:**

It is still controversial whether primary tumor resection (PTR) improves survival in colorectal cancer (CRC) patients with unresectable metastases.

**Methods:**

Colon cancer patients were enrolled and randomly allocated to with or without PTR after induction chemotherapy with XELOX or mFOLFOX6, and those with chemotherapy failure were excluded. The primary endpoint was TTF (time to strategy failure) on an intent-to-treat basis. This study is registered with ClinicalTrials.gov, number NCT02291744.

**Results:**

Between April 2015 and July 2020, 140 patients were enrolled, and 54 patients were excluded due to colon obstruction (16), perforation (1), disease progression (22), death (1), radical resection (3), or other reasons (11). After induction chemotherapy, 86 patients were randomized into group A (the resection group, n = 42) or group B (chemotherapy-alone group, n = 44). The median TTF was 143 days (95% CI: 104.9–181.1) in group A and 196 days (95% CI: 96.5–295.5) in group B (HR: 0.930 95% CI: 0.589–1.468, p = 0.755), and there was no significant difference in PFS, OS, and incidence of chemotherapy-related adverse events between two groups. The primary lesion-related events after PTR in group A were significantly fewer than those in group B. Patients with a tumor regression grade (TRG) score of 2 had longer TTF and PFS than those with score of 3.

**Conclusion:**

PTR after induction chemotherapy could not bring survival benefits for colon cancer patients with unresectable metastases, and it is not recommended routinely. However, it also requires individualized treatment as colon obstruction or perforation occurred in some patients and PTR could reduce primary tumor-related events, and the TRG score might help for selection of beneficial patients.

## Introduction

Colorectal cancer (CRC) is the third leading cause of cancer-related death in males and the second most common cause of cancer-related death in females (1, 2). At the time of diagnosis, approximately 15%~25% of patients already have distant metastasis, of which 80% are unresectable (3). For CRC patients with resectable metastases, surgical resection is the best treatment, achieving a 5-year survival rate of 30% (4). For patients with unresectable metastases, NCCN and ESMO guidelines have reached a consensus that palliative primary tumor resection (PTR) should be considered when there is a risk of bleeding, obstruction or perforation. However, it is still controversial whether PTR is needed when the aforementioned symptoms are absent.

From the perspective of those who support PTR, surgery can prevent tumor perforation, bleeding, colon obstruction, and other complications during chemotherapy. Once these complications occur, the mortality rate of emergency surgery is very high due to the effects of chemotherapy on systemic conditions, bone marrow function, and immunity. In addition, surgery can alleviate the tumor load of patients, which may increase treatment response and bring survival benefits. The opposite view was that surgery delays the onset of systemic treatment, and its own impact on the physical condition might lead to rapid progression of the tumor. Besides, surgical complications may affect subsequent systemic treatment, while effective chemotherapy can achieve a higher response rate and disease control rate. Therefore, for CRC patients with asymptomatic primary lesion and synchronous unresectable metastases, whether the primary tumor increases the risk of gastrointestinal complications during subsequent treatment and whether PTR brings additional survival benefits are two concerns for consideration.

Retrospective studies have found that the overall risk of complications that require surgical intervention caused by primary tumor was generally low (less than 15%) and systemic chemotherapy was safe as an initial treatment, while PTR did not decrease the risk of gastrointestinal complications (5–7). Some retrospective studies suggested that initial PTR did not benefit patients’ survival and increased the risk of death conversely (8). There were also many studies suggesting that initial PTR may benefit patients (9–13). However, in these retrospective studies, there were significant differences in baseline data which might lead to differences of eventual survival results. Hence, prospective randomized controlled studies are imperative.

Most recently, there were 2 reports of prospective randomized clinical trials to compare the effects of PTR followed by chemotherapy with chemotherapy alone, but the results in these 2 studies are also different and controversial (14, 15). We also performed this randomized phase II trial from 2015 in colon cancer patients with unresectable metastases, but the design of our study was different with the two reported studies, as we enrolled patients and gave them induction chemotherapy for 3 months, excluded patients with chemotherapy failure whose prognosis was always poor, and then randomly divided them into PTR group and chemotherapy group to compare the effects of induction chemotherapy followed by PTR and chemotherapy alone and determine the value of PTR after induction chemotherapy.

## Methods

### Patients

Colon cancer patients with asymptomatic primary lesion and synchronous unresectable metastases were eligible for this study, and additional key inclusion criteria were as follows: age between 18 and 80 years old; Eastern Cooperative Oncology Group (ECOG) performance status (PS) of 0–1; histologically confirmed colon adenocarcinoma; at least one measurable lesion according to Response Evaluation Criteria in Solid Tumors (RECIST version 1.1); had not received chemotherapy for metastatic disease; adequate bone marrow, liver, and renal function; and patients voluntarily agreed to participate this clinical trial. Key exclusion criteria included intestinal perforation, bleeding, and ileus that require surgical intervention; multiple primary colon cancer; with brain or meningeal metastases; had vital organ failure or other serious diseases; malignant peritoneal effusion; patients had indications for the application of targeted therapy, and could economically afford them. Full eligibility criteria are provided in the protocol (**Supplement 1**).

This trial complies with the International Ethical Guidelines for Biomedical Research Involving Human Subjects and the Declaration of Helsinki. All patients were informed and signed written informed consent before enrollment. The trial protocol was approved by the medical ethics committee of Fudan University Shanghai Cancer Center.

### Study Design and Procedures

This is an open-label, single-center, prospective, randomized phase II trial in China (registered with ClinicalTrials.gov, number NCT02291744). Colon cancer patients with unresectable metastases at enrollment will be randomly allocated to either resection group (group A) or chemotherapy alone group (group B), after receiving induction chemotherapy with 4 cycles of XELOX (oxaliplatin 130 mg/m (2) intravenously on day 1; capecitabine 1,000 mg/m (2) p.o. bid from day 1 to day 14; repeated every 3 weeks) or 6 cycles of mFOLFOX6 regimen (oxaliplatin 85 mg/m (2), Leucovorin 400 mg/m (2), and fluorouracil 400 mg/m (2) intravenously on day 1, followed by a 46-h continuous infusion of fluorouracil 2,400 mg/m (2); repeated every 2 weeks), excluding those with disease progression, primary lesion failure (colon perforation, bleeding, or ileus which needs local intervention), lesions becoming radically resectable, or primary lesion unresectable. After randomization, patients in group A received PTR and then continued the original regimen chemotherapy after surgery (3–4 weeks) with 4 cycles of XELOX or 6 cycles of mFOLFOX6 regimen as consolidation chemotherapy, followed by capecitabine maintenance chemotherapy; patients in group B continued 4 cycles of XELOX or 6 cycles of mFOLFOX6, and capecitabine maintenance afterward. During maintenance therapy, if progression occurs within 3 months after discontinuation of oxaliplatin, the second-line regimen will be performed; if progression occurs beyond 3 months after discontinuation of oxaliplatin and toxicity has recovered to grade I, the original regimen will be applied again until second progression or intolerable toxicity.

### Randomization and Masking

Eligible patients were randomly assigned (1:1) to either PTR or chemotherapy group *via* a computer-generated randomization schedule managed by King Yee Company (Beijing, China) after induction chemotherapy and stratified by the number of metastatic sites (1 or more) and tumor response (PR or SD) after 2 evaluations. The patients, investigators, and study team were not masked to the allocated treatment as this study was open-label.

### Outcomes

The primary endpoint was time to failure of strategy (TTF, defined as the time from randomization to secondary progression in patients that receive reintroduction of the original induction chemotherapy regimen after maintenance therapy, or to the first progression in patients without reintroduction of the original regimen, or to the time of primary lesion failure including colon perforation, bleeding, or ileus which needs local intervention). The secondary endpoints included progression-free survival (PFS, defined as the time from randomization to first progression or death from any cause, whichever occurred first), overall survival (OS, defined as the time from enrollment to death), adverse events (AEs), objective response rate (ORR), surgical complications, and proportion of surgical interventions for primary lesion in group B.

Tumors were assessed radiologically at baseline, every 6 weeks during induction chemotherapy and consolidation chemotherapy, and every 8 weeks during maintenance therapy. The tumor response was evaluated according to RECIST 1·1 criteria. For patients in group A, the resected primary tumor tissues were used to evaluate the pathological response to induce chemotherapy according to tumor regression grade (TRG) score (16). Adverse events (AEs) were graded according to National Cancer Institute Common Terminology Criteria for Adverse Events (NCI-CTCAE version 4.0).

### Statistical Analysis

This study was designed to assess the superiority of induction chemotherapy followed by PTR compared with chemotherapy alone in terms of TTF. A number of previous phase III trials have shown that the mPFS of first-line chemotherapy for mCRC was about 8 months. As patients received a total of 3-month induction chemotherapy in this trial, after randomization, the median TTF (mTTF) might be about 5 months in the chemotherapy-alone group (group B), and it might be longer in the surgical group (group A) as PTR might reduce the rate of primary lesion failure. The mTTF was assumed to be 5 months in group B and 9 months in group A, which get the clinical valuable absolute benefit of 4 months prolonged, with a corresponding hazard ratio (HR) of 0.556. Based on HR = 0.8, α = 0.05, and 20% dropout rate, a total of 130 patients (number of cases after randomization) are required in this study.

An assessment of efficacy was performed mainly in the intent-to-treat (ITT) set which included all the patients who had signed the informed consent and underwent randomization, and in the per-protocol (PP) set which included patients with no serious violations of the study protocol as supplement. Patients who had received at least one cycle of chemotherapy (3 patients in group A and 1 patient in group B were excluded because they did not receive chemotherapy after randomization), and at least one safety evaluation after randomization was included in the safety set (SS). On that basis, what should be specifically stated was that 1 patient randomly assigned to group A received chemotherapy alone without PTR which was included in group B of the modified SS for safety analysis, and similarly, 1 patient who had been assigned to group B received PTR after randomization which was included in group A for safety analysis. Modified SS served as the primary population for safety analyses in this study.

Survival curves were estimated according to the Kaplan–Meier method and compared by the log-rank test. The Cox proportional hazards model was used to calculate hazard ratios (HRs) and 95% confidence intervals (CIs). Enumeration data were compared using the χ (2) test. All hypothesis tests used in this study were two-sided tests with 0·05 as the test level. All statistical analyses except for survival analysis were performed using the Statistical Package for the Social Sciences, version 24·0 (SPSS, Inc.). R packages called “survival” and “survminer” were used for survival analysis through R software version 3·6·0 (https://www.r-project.org/).

## Results

**Figure 1:**
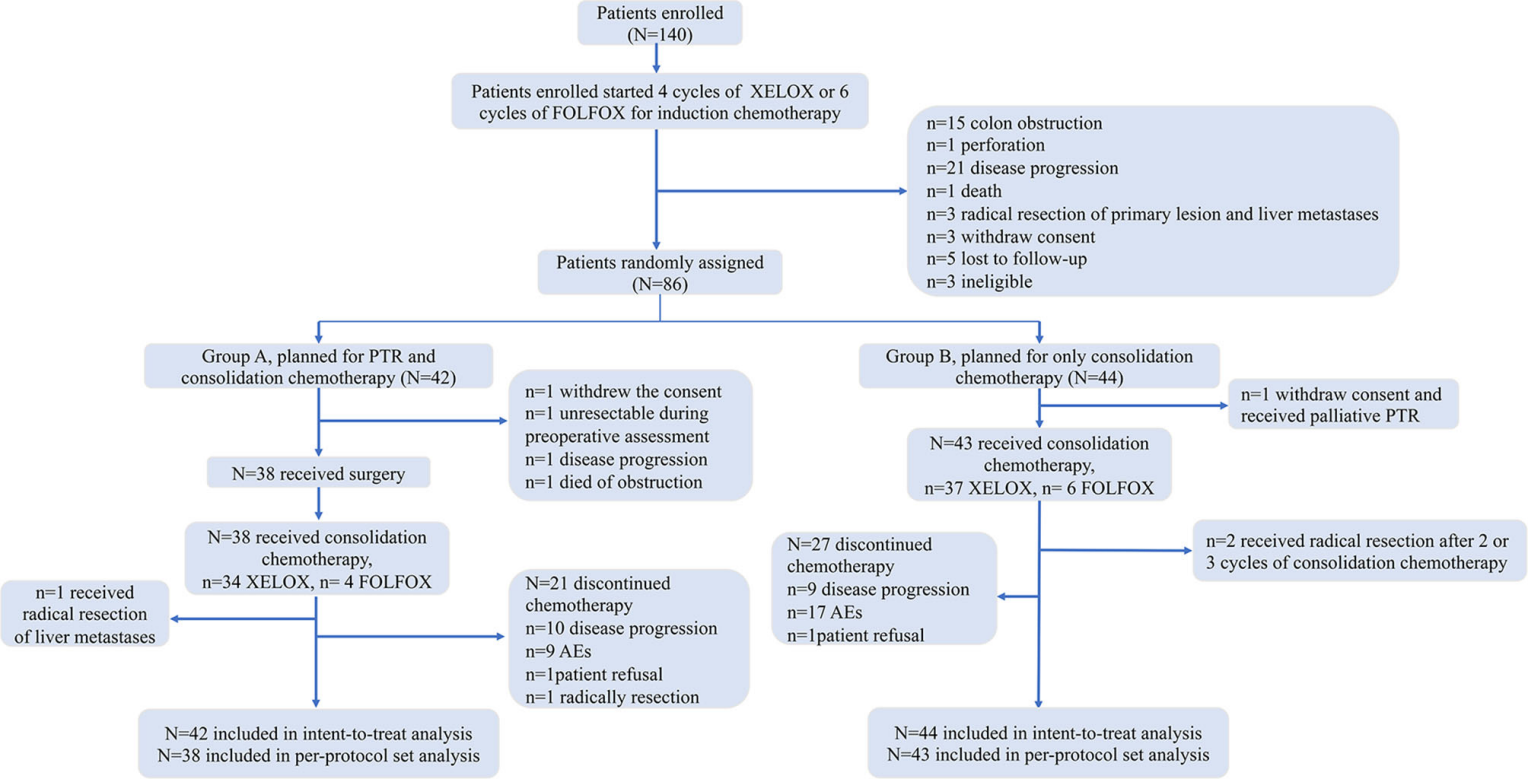
CONSORT diagram.

Between April 3, 2015, and July 16, 2020, 140 patients were enrolled, and we terminated the trial in advance, as the negative result of PTR in the JCOG1007 study was reported in the 2020 CSCO meeting. Before randomization, 54 patients were excluded due to colon obstruction (15), perforation (1), disease progression of metastatic lesions (21), death (1), radical resection of primary lesion and liver metastases (3), withdrawal of consent (3), loss to follow-up (5), and ineligibility (3). Finally, 86 patients (51 patients achieved PR, 35 achieved SD) were randomly grouped into group A (n = 42) or group B (n = 44). Four patients randomly assigned to group A did not receive surgery, including 1 who withdrew consent, 1 who died of obstruction, 1 who had disease progression, and 1 with unresectable primary lesions (defined unresectable during preoperative assessment). There were 22 patients who underwent laparoscopic surgical resection, and the other 16 patients received the open surgery. One patient randomly assigned to group A received radical resection of liver metastases after PTR and 2 cycles of consolidation chemotherapy. For patients randomly assigned to group B, 1 withdrew consent and received palliative PTR and 2 received radical resection of the primary and metastatic lesions after 2 or 3 cycles of consolidation chemotherapy. All the data above are shown in **Figure 1**.

The baseline clinical–pathological characteristics between two groups were well balanced (**Table 1**). The median treatment duration (induction plus consolidation chemotherapy) was 7 cycles (ranging 4–8, 95% CI: 6·21–7.07 cycles) of XELOX or 10 cycles (ranging 8–11, 95% CI: 5.87–13.46 cycles) of mFOLFOX6 in group A, and 6 cycles (ranging 4–8, 95% CI: 6.33–7.05 cycles) of XELOX or 10 cycles (ranging 9–12, 95% CI: 8.77–11.56 cycles) of mFOLFOX6 in group B. Patients did not complete planed cycles of chemotherapy due to disease progression (14 in group A and 9 in group B), AEs (10 in group A and 17 in group B), patient refusal (2 in group A and 2 in group B), or radical resection (1 in group A). 13 patients (31%) in group A and 18 patients (41%) in group B suffered a dose reduction due to the toxicity of chemotherapy.

**Table 1:** Demographics and baseline characteristics.

Factors	A (n = 42)	B (n = 44)
**Age (years)**	57 (32–73)	60 (22–73)
**Sex**		
Male	26 (62%)	25 (57%)
Female	16 (38%)	19(43%)
**Pathological**		
Adenocarcinoma	37 (88%)	42 (95%)
Mucinous adenocarcinoma	5 (12%)	2 (5%)
**Chemotherapy regimen**		
Xelox	38 (90%)	38 (86%)
Folfox	4 (10%)	6 (14%)
**Location of primary tumor**		
Right-half	16 (38%)	17 (39%)
Left-half	26 (62%)	27 (61%)
**Location of metastasis**		
Liver	16 (38%)	13 (30%)
Lung	1 (2%)	3 (7%)
Multiple	25 (60%)	28 (63%)
**KRAS**		
Wild type	13 (31%)	14 (32%)
Mutation	19 (45%)	17 (39%)
Unknown	10 (24%)	13 (30%)
**NRAS**		
Wild type	31 (74%)	31 (70%)
Mutation	1 (2%)	0 (0)
Unknown	10 (24%)	13 (30%)
**BRAF**		
Wild type	30 (71%)	30 (68%)
Mutation	2 (5%)	1 (2%)
Unknown	10 (24%)	13 (30%)

### Efficacy

For all the enrolled 140 patients, 129 patients were assessable for tumor response, and 64 patients achieved partial response (PR), 56 stable disease (SD), and 9 progression of disease (PD), with an overall response rate (ORR) of 49.6%.

**Figure 2:**
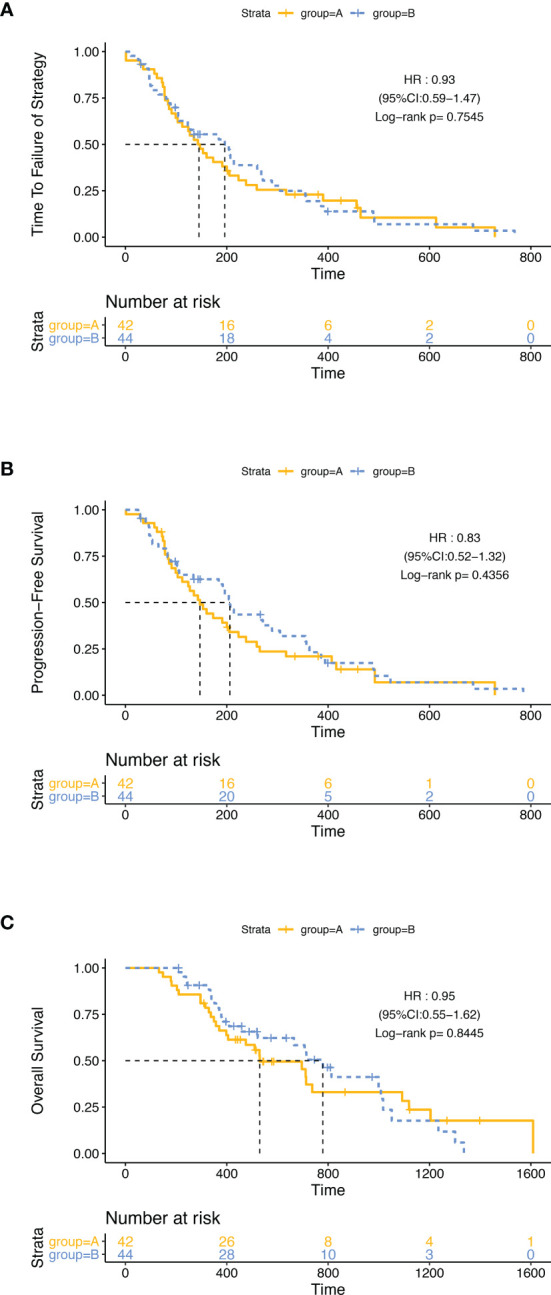
Kaplan–Meier curves in the intention-to-treat population. The differences of TTF **(A)**, PFS **(B)**, and OS **(C)** between group A and group B were not significant.

At the time of data cutoff (February 10, 2021), 55 (64·0%) of 86 patients had died; 75 cases (87·2%) had a failure of strategy, and 72 cases (83·7%) had disease progression. The median follow-up time was 464 days, ranging from 132 to 1,609 (95% CI: 432.88–658.30). In the ITT analysis, 37 (88.1%) of 42 patients in group A and 38 (86.4%) of 44 patients in group B had a failure of strategy, and the mTTF was 143 days (95% CI: 104.9–181.1) in group A and 196 days (95% CI: 96.5–295.5) in group B (HR: 0.930 95% CI: 0.589–1.468, p = 0·755). 35 (83.3%) of 42 patients in group A and 37 (84.1%) of 44 patients in group B had disease progression. The mPFS was 147 days (95% CI: 105.7–188·3) in group A and 206 days (95% CI: 180.9–231.1) in group B (HR: 0.831, 95% CI: 0.522–1.323, p = 0.436). 28 (66.7%) of 42 patients in group A and 27 (61.4%) of 44 patients in group B had died. The mOS was 530 days (95% CI: 308.9–751.1) in group A, and 779 days (95% CI: 626.3–931.7) in group B (HR: 0.948 95% CI: 0.554–1.622, p = 0·845). There was no significant difference in TTF, PFS, and OS in the two groups (**Figure 2**).

In PP set analysis, 4 patients who did not receive surgery in group A and 1 patient who withdrew consent in group B were excluded, and there was also no significant difference in TTF, PFS, and OS between group A and group B (**Figure S1**).

**Figure 3:**
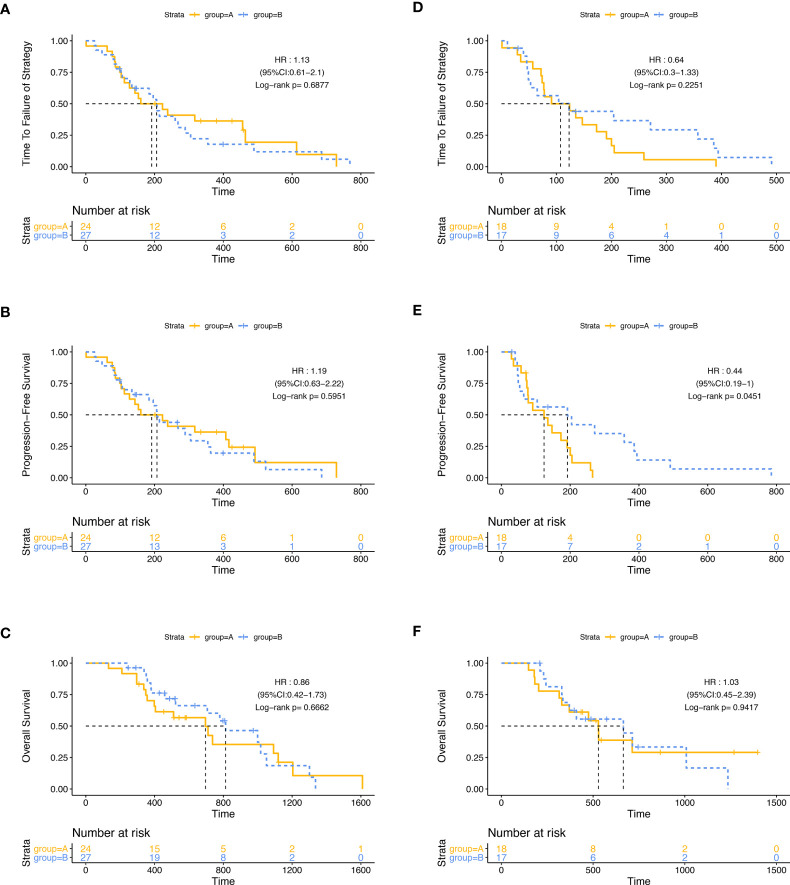
Subgroup analysis. The differences of TTF **(A)**, PFS **(B)**, and OS **(C)** between group A and group B were not significant among patients who achieved PR. Among patients who achieved SD, the differences of TTF **(D)** and OS **(F)** between group A and group B were not significant; patients in group B had a longer PFS **(E)**.

**Figure 4:**
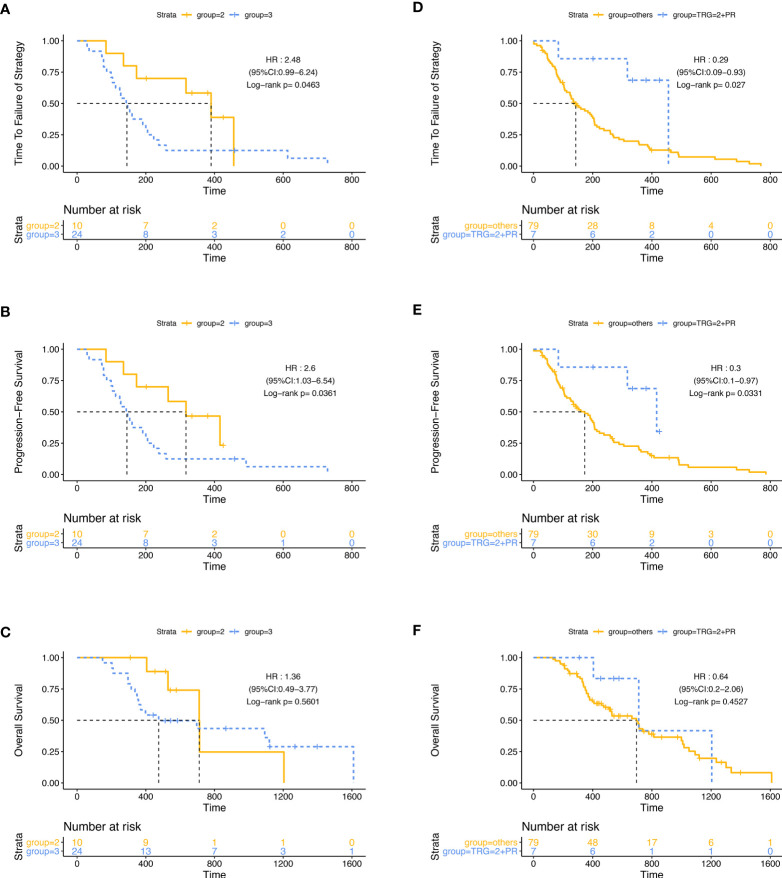
Stratification analysis with the TRG score in group A. Patients with the TRG score of 2 had longer TTF **(A)** and PFS **(B)** but similar OS **(C)** than patients with a TRG score of 3. Patients with a TRG score of 2, and meanwhile achieved PR had longer TTF **(D)** and PFS **(E)** but similar OS **(F)** than others.

Then, we did a subgroup analysis in the ITT population. Firstly, we grouped the 86 patients by the best efficacy (PR or SD) and found that patients who achieved PR had a longer TTF and PFS than those with SD (**Figure S2**). In the subgroup of patients with SD, the mPFS of patients in group A (124 days, 95% CI: 48.2–199.8) was significantly shorter than that in group B (192 days, 95% CI: 15.1–368.9; p = 0·045, HR: 0.436, 95% CI: 0.189–1.002), while in patients with PR, PFS was similar between two groups, and there was no significant difference in mTTF and mOS between groups A and B, either in the patients with PR or in patients with SD (**Figure 3**). Furthermore, we stratified the patients in group A by the tumor regression grade (TRG) score, which were available in 34 patients, and found that compared with patients with the TRG score of 3 (24/34,70.6%), the patients with a TRG score of 2 (10/34, 29.4%) had longer mTTF (TRG score = 2: 390 days, 95% CI: 249.0–531.0; TRG score = 3: 143 days, 95% CI: 108.2–177.8; p = 0·046, HR: 0.403, 95% CI: 0.16–1.014) and mPFS (TRG score = 2: 317 days, 95% CI: 176.8–457.2, TRG score = 3: 143 days, 95% CI: 108.2–177.8; p = 0.036, HR: 0.386, 95% CI: 0.153–0.970) **Figures 4A, C**). Moreover, we also found that patients with TRG score of 2 and meanwhile achieved PR had longer mTTF and mPFS than others (**Figures 4D–F**).

### Safety

Among the patients in group A, major surgery-related complications were as follows: 1 patient had liver abscess, 5 patients had increased ALT or AST, and 2 patients had a fever after surgery. For chemotherapy-related AEs (**Table 2**), hematological AEs like leucopenia, neutropenia, thrombocytopenia, and anemia were proved with no significant difference between two groups; so were the non-hematological AEs like diarrhea, anorexia, nausea, and hand–foot syndrome.

**Table 2:** Hematological and non-hematological adverse events associated with chemotherapy (mSS set).

	Any grade			Grade 3/4		
	A (n = 39)	B (n = 43)	p value	A (n = 39)	B (n = 43)	p value
**Hematological**						
Leucopenia	14 (35.9)	24 (55.8)	0.071	2 (5.1)	3 (7.0)	1.000
Neutropenia	24 (61.5)	29 (67.4)	0.577	7 (17.9)	2 (4.7)	0.116
Thrombocytopenia	30 (76.9)	31 (72.1)	0.617	10 (25.6)	12 (27.9)	0.817
Anemia	23 (59)	24 (55.8)	0.773	2 (5.1)	0 (0)	0.223
**Non-hematological**						
ALT abnormality	13 (33.3)	15 (34.9)	0.882			
AST abnormality	25 (64.1)	32 (74.4)	0.311	2 (5.1)	1 (2.3)	0.931
Diarrhea	6 (15.4)	6 (14.0)	0.855	1 (2.6)	1 (2.3)	1.000
Anorexia	11 (28.2)	12 (27.9)	0.976			
Nausea	14 (35.9)	11 (25.6)	0.311			
Vomiting	8 (20.5)	6 (14.0)	0.43			
Hand–foot syndrome	3 (7.7)	8 (18.6)	0.148	0 (0)	1 (2.3)	1.000
Fatigue	9 (23.1)	4 (9.3)	0.088			
Peripheral sensory neuropathy	15 (38.5)	25 (58.1)	0.075	0 (0)	1 (2.3)	1.000

Data are n (%).

ALT, alanine aminotransferase; AST, aspartate aminotransferase.

### Primary Lesion-Related Events

After randomization, primary lesion-related events had occurred more often in group B, such as obstruction (4 in group B (9·1%) and none in group A), bloody stools (5 (11.4%) in group B (including 2 patients with Hgb less than 80 g/l), and 1 (2.4%) in group A), and 12 patients (27·3%) with at least 2+ occult blood in stools found in group B while none was found in group A. The incidence of obstruction and bloody stools in group A was significantly lower than that in group B (2.5% vs. 20.5%, p = 0·028). There were 4 (9.1%) patients received local interventions for primary lesion related events in group B, including 1 patient who received PTR because of colon obstruction after 4 cycles of consolidation chemotherapy, 1 patient who received intestinal stent placement because of colon obstruction, 1 patient who received PTR because of bloody stools after 4 cycles of consolidation chemotherapy, and 1 who received laparotomy after 1 cycle of consolidation chemotherapy because of bloody stools but with primary tumor unresectable found during operation. We also found that 9 patients in group A suffered anemia with Hgb less than 80 g/l which might partially be due to bloody stools (with positive occult blood in stools) before randomization and 6 (66.7%) of them recovered (Hgb increased more than 20 g/l) after PTR, while only 1 (20%) of 5 patients in group B recovered after randomization.

## Discussion

For CRC patients with unresectable metastases, whether PTR could have a favorable impact on survival has been controversial. Sabine performed a retrospective analysis (9), which included two studies: in the CAIRO study, 258 patients had undergone PTR and 141 patients had been treated with chemotherapy; in the CAIRO2 study, 289 patients had undergone PTR and 159 patients had been treated with chemotherapy and targeted therapy. In the CAIRO study, the median OS and PFS of the resection group were 16.7 and 6.7 months, respectively, significantly better than 11.4 and 5.9 months in the non-resection group. In the CAIRO2 study, the median OS and PFS of the resection group (20.7 and 10.5 months) were also significantly better than those of the non-resection group (13.4 and 7.8 months). Aslam et al. reported their 10-year follow-up data of 920 CRC patients with unresectable metastases; the results showed prolonged median survival in the resection group (14.5 months) compared to the non-resection group (5.83 months) (10). A meta-analysis (11) retrieved data of 1,155 CRC patients with unresectable metastases including four independent randomized controlled trials about first-line therapy (FFCD-9601, FFCD-2000-05, ACCORD-13, and ML-16987) to evaluate the impact of PTR on survival. Among 810 patients who met the inclusion criteria, 478 patients (59%) underwent PTR and had significantly prolonged survival. In the multivariate analysis, PTR was independently associated with better overall survival and PFS. A meta-analysis incorporating 26 studies showed that among 43,903 patients with CRC, 29,639 cases receiving PTR combined with chemotherapy/radiotherapy had longer overall survival (HR = 0.59, [0.51–0.68]) and PFS (HR = 0.73, [0.58–0.91]) (12). These findings indicated that PTR could be beneficial to survival. However, it should be pointed out that patients in the resection group were often those with lower tumor load and better general condition, so the selection bias might lead to the difference in survival. Alawadi et al. performed an observational cohort study of 15,154 patients with unresectable metastatic colon cancer in which 8,641 patients underwent PTR and found that PTR was associated with a significant reduction in mortality; however, after adjustment for confounder effects, PTR was not associated with improved survival compared with systemic chemotherapy (13).

Most recently, a prospective randomized trial with a very small sample size (48) of participants was reported, and the results showed that, compared with chemotherapy alone, PTR followed by chemotherapy improved the 2-year cancer-specific survival of patients with asymptomatic stage IV CRC, although no statistical difference was obtained, which might due to the limited patients (14). However, different results were reported in another prospective randomized trial (JCOG1007 study) with a larger sample size of participants which enrolled 165 patients (15). In this trial, PTR did not bring survival benefit but brought complications, including 3 postoperative deaths and decreased tolerance to chemotherapy. In addition, it was found that only 13% of the patients in the chemotherapy-alone group received subsequent surgical primary lesion intervention. The results suggested that PTR is not necessary generally, the proportion of patients who need PTR during chemotherapy is not high, and initial PTR could not bring survival benefits. These two studies both evaluated the value of initial PTR, in which delayed chemotherapy by PTR might bring survival damage for patients with excessive tumor burden. In fact, these patients need chemotherapy to achieve rapid tumor control. The existence of these patients may counteract or reduce the effects of the benefit subpopulation which may exist in the PTR group, thus resulting in no overall benefits. Therefore, the operation opportunity and the selection of the benefit subpopulation should be considered. The design of our study was different from these two reported trials. In our study, patients received induction chemotherapy firstly, and then the patients with no disease progression were randomized into PTR or control group. The design of induction chemotherapy followed by PTR, which is different from the JCOG1007 study, might have two aspects of advantages. On the one hand, it could exclude the patients who do not respond to chemotherapy or have rapid tumor progression. These patients always have poor prognosis and short survival and are difficult to benefit from PTR. On the other hand, effective induction chemotherapy could reduce tumor burden, and improve prognosis, thus bringing possibility for patients to benefit from PTR.

We enrolled 140 patients, and 86 patients were randomly divided into two groups after induction chemotherapy. There was no difference in TTF, PFS, and OS between two groups, which showed that PTR after induction chemotherapy also could not prolong survival for colon cancer patients with unresectable metastases, so PTR was not recommended routinely. However, different from the results of the JCOG1007 study, the complications of PTR were relatively slight, and no postoperative deaths occurred in our study. Furthermore, PTR reduced primary tumor-related events which might help to improve quality of life for some patients. During induction chemotherapy, 12.1% (17/140) of patients experienced obstruction or perforation, and during consolidation and maintenance chemotherapy after randomization, 9.1% (4/44) of patients received local interventions for primary lesion-related events in the chemotherapy-alone group, so individualized treatment was required. Subgroup analysis showed in SD patients that the mPFS of the PTR arm was shorter than that of the chemotherapy arm. The possible reason was that in the PTR group, chemotherapy was discontinued due to surgery and then the metastatic lesions were more likely to progress during this period, while in PR patients, the mPFS of the PTR group was not shortened, suggesting that the PFS of PR patients was relatively long, and the suspension of chemotherapy due to PTR did not affect the PFS. Therefore, for SD patients after induction chemotherapy, PTR was not recommended, while for PR patients after induction chemotherapy, surgery did not shorten the PFS and could reduce the primary tumor-related events which might help to improve the life quality. In the future, it was worth expanding the sample size to explore the benefits brought by PTR in this part of patients. As only chemotherapy drugs were used in this study, the addition of molecular target drugs to further improve the remission rate and prolong PFS might increase the benefit and reduce the primary tumor-related events during a longer period of metastatic lesion progression-free survival.

Moreover, patients with a low TRG score (primary tumor regression was relatively good) also had longer mPFS and mTTF, which were consistent with the results of longer mPFS and mTTF in patients whose efficacy was evaluated as PR (mainly from the regression of metastatic lesions), which might also be helpful to screen patients who might truly get benefit in future studies. It should be noted that the TRG score was derived from surgically resected specimens, and information could not be obtained before surgical resection. However, in the future, the TRG score might be evaluated by colonoscopy biopsy after induction chemotherapy.

## Data Availability Statement

The original contributions presented in the study are included in the article/**Supplementary Material**. Further inquiries can be directed to the corresponding authors.

## Ethics Statement

The studies involving human participants were reviewed and approved by the medical ethics committee of Fudan University Shanghai Cancer Center. The patients/participants provided their written informed consent to participate in this study.

## Author Contributions

MH, YY, and QL contributed equally to this work as first authors. MH and YY had full access to all of the data in the study and take responsibility for the integrity of the data and the accuracy of the data analysis. WG and XL did concept and design. All the authors did the acquisition, analysis, or interpretation of data. YY and WG drafted the manuscript and did the critical revision of the manuscript for important intellectual content. YY did the statistical analysis. DH, XS, and WG gave administrative, technical, or material support. WG supervised the manuscript. All authors contributed to the article and approved the submitted version.

## Funding

This trial was supported by the funding from the Fudan University Shanghai Cancer Center.

## Conflict of Interest

The authors declare that the research was conducted in the absence of any commercial or financial relationships that could be construed as a potential conflict of interest.

## Publisher’s Note

All claims expressed in this article are solely those of the authors and do not necessarily represent those of their affiliated organizations, or those of the publisher, the editors and the reviewers. Any product that may be evaluated in this article, or claim that may be made by its manufacturer, is not guaranteed or endorsed by the publisher.

## Supplementary Material

Supplementary Figure S1 Kaplan-Meier curves in the Per-protocol population. The differences of TTF **(A)**, PFS **(B)** and OS **(C)** between group A and group B were not significant.

Supplementary Figure S2 Kaplan-Meier curves according to tumor response (PR vs. SD). Patients achieved PR had longer TTF **(A)**,PFS **(B)**, but similar OS **(C)** than those achieved SD.

Supplement 1 Clinical trial protocol.
