# Supplementary material for: Induction Chemotherapy Followed by Primary Tumor Resection Did Not Bring Survival Benefits in Colon Cancer Patients With Asymptomatic Primary Lesion and Synchronous Unresectable Metastases
Source: Front Oncol. 2022 Jan 31;12:747124. doi: 10.3389/fonc.2022.747124 (PMC8841852; doi:10.3389/fonc.2022.747124)
Supplement: Supplementary Figure S1 — Kaplan-Meier curves in the Per-protocol population. The differences of TTF (A), PFS (B) and OS (C) between group A and group B were not significant. [file DataSheet_1.pdf]

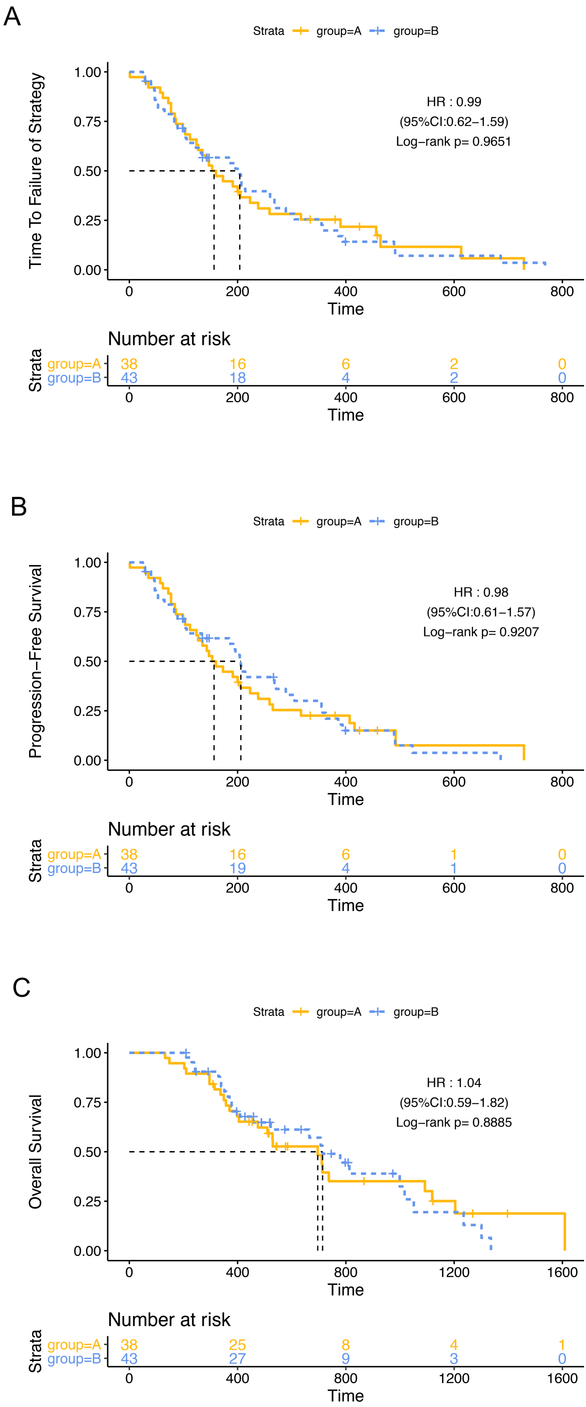

Figure S1 Kaplan-Meier curves in the Per-protocol population. The differences of TTF (A), PFS (B) and OS (C) between group A and group B were not significant.
