# Supplementary material for: Induction Chemotherapy Followed by Primary Tumor Resection Did Not Bring Survival Benefits in Colon Cancer Patients With Asymptomatic Primary Lesion and Synchronous Unresectable Metastases
Source: Front Oncol. 2022 Jan 31;12:747124. doi: 10.3389/fonc.2022.747124 (PMC8841852; doi:10.3389/fonc.2022.747124)
Supplement: Supplementary Figure S2 — Kaplan-Meier curves according to tumor response (PR vs. SD). Patients achieved PR had longer TTF (A),PFS (B), but similar OS (C) than those achieved SD. [file DataSheet_2.pdf]

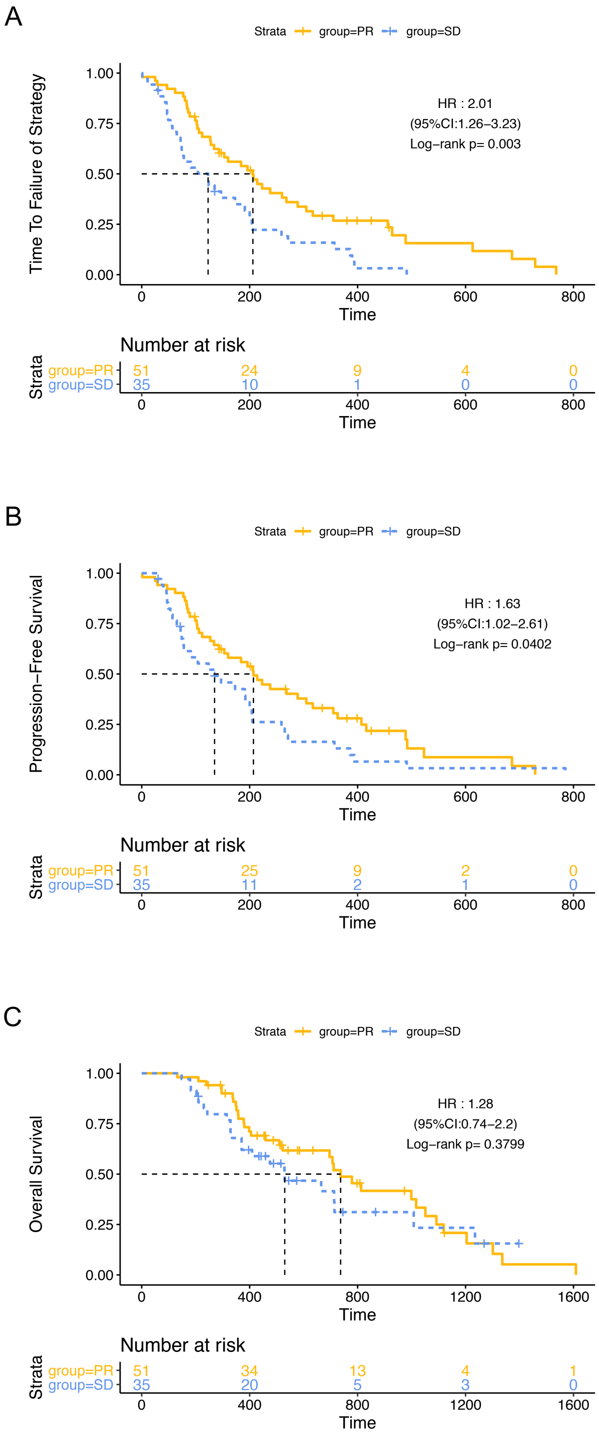

Figure S2 Kaplan-Meier curves according to tumor response (PR vs. SD). Patients achieved PR had longer TTF (A) ,PFS (B), but similar OS (C) than those achieved SD.
