# Supplementary material for: Induction Chemotherapy Followed by Primary Tumor Resection Did Not Bring Survival Benefits in Colon Cancer Patients With Asymptomatic Primary Lesion and Synchronous Unresectable Metastases
Source: Front Oncol. 2022 Jan 31;12:747124. doi: 10.3389/fonc.2022.747124 (PMC8841852; doi:10.3389/fonc.2022.747124)
Supplement: Supplement 1 — Clinical trial protocol. [file DataSheet_3.doc]

Supplement 1

Comparison of XELOX or mFOLFOX6 regimen chemotherapy combined with vs without surgical resection of the primary lesion for colon cancer with unresectable metastases: A randomized, controlled, phase II clinical study

**PI:** Prof. Weijian Guo

Fudan University Shanghai Cancer Center

**Protocol Number: FDM201404GI**

Protocol Version: 6.0

Protocol Date: December 5, 2016

（First Version date: Jan, 2015)

This trial will be conducted in strict compliance with the protocol, GCP and local regulations.

1. Background

**Background**:Colorectal cancer (CRC) whose incidence rate ranks third in male malignancies and second in female malignancies is one of the most common gastrointestinal cancer and there are currently more than 1.2 million new cases and more than 600,000 patients die of it worldwide each year. At the time of diagnosis, approximately 15% ~ 25% of the patients already have distant metastasis, and about 80% of patients with metastasis have unresectable metastases, which is also the leading cause of death in CRC patients.

For CRC patients with resectable metastases, surgical resection is the best treatment method, as the five-year survival rate after surgery is 30%. Most patients have unresectable metastases when diagnosed. NCCN and ESMO guidelines have reached a consensus on the primary tumor treatment of those patients that palliative surgical resection of the primary tumor should be considered when the primary tumor has the risk of bleeding, obstruction or perforation. However, it is still controversial whether to resect the primary tumor when there is no bleeding, obstruction or perforation in the primary tumor. NCCN guidelines point out that palliative resection of the primary tumor should not be performed routinely when the primary tumor has no obvious and severe symptoms. ESMO guidelines suggest that for initially asymptomatic patients with lesions that progress slowly relatively, systemic chemotherapy should be taken as the primary therapeutic method, and watchful waiting can be adopted for the primary tumor.

From the perspective which supports surgical resection of the primary tumor, surgery can prevent tumor perforation, obstruction, bleeding and other complications during chemotherapy. Once these complications occur, the mortality rate of emergency surgery is very high due to the effects of chemotherapy on systemic conditions, bone marrow function and immunity. In addition, surgery can alleviate the tumor load of patients, which may increase the treatment response and bring survival benefits. The opposite view is that surgery delays the onset of systemic treatment, and its own impact on the physical condition can lead to rapid progression of the tumor. Besides, complications of the surgery affect the subsequent systemic treatment, while effective chemotherapy can achieve higher response rates and disease control rates. Therefore, for colorectal cancer with unresectable metastases and primary tumor without obvious symptoms, whether the primary tumor increases the risk of gastrointestinal complications during subsequent treatment and whether surgical resection of the primary tumor brings additional survival benefits are the two questions the debate on whether the primary tumor should be resected focused on.

Recently many studies have found that chemotherapy does not increase the risk of gastrointestinal complications. Nitzkorski reported that 103 patients with metastatic CRC took 5-FU as the initial treatment and oxaliplatin, irinoticam or bevacizumab were used among the patients who developed symptoms. The incidence of local complications caused by primary tumor was 9.8%. Besides, age, gender, metastatic sites and type of chemotherapy were not risk factors for complications. Tebbutt conducted a retrospective analysis of 362 asymptomatic mCRC patients with 82 cases in chemotherapy group and 280 cases in surgery group. Initial chemotherapy did not increase the incidence of local complications such as intestinal obstruction, peritonitis, fistula and intestinal bleeding, while peritoneal metastasis is the main cause of intestinal obstruction and surgical resection cannot prevent or reduce the obstruction caused by peritoneal metastasis. Another retrospective study conducted by Seo GJ of 283 asymptomatic stage IV CRC patients indicated that there was no difference between the incidence of gastrointestinal complications after surgery and chemotherapy. Surgical resection could not reduce the incidence of intestinal obstruction, bleeding, peritonitis, and intestinal fistula. For asymptomatic mCRC patients, chemotherapy did not increase the local complications caused by the primary tumor, and resection did not reduce the local complications. Therefore, based on the evidence from the current retrospective studies , the primary tumor does not increase the risk of gastrointestinal complications during subsequent treatment. The overall risk of complications that require surgical intervention caused by primary tumor is generally less than 15% and systemic chemotherapy is safe as an initial treatment, while surgical resection of the primary tumor does not decrease the risk of gastrointestinal complications.

Whether surgical resection of the primary tumor brings additional survival benefits is still controversial. Some studies suggested that initial surgical resection of the primary colorectal cancer did not benefit the patients’ survival and it increased the risk of death conversely [9]. There were also many studies suggesting that initial resection of the primary colorectal cancer may benefit patients. Significantly, in these retrospective studies, there were significant differences in baseline data between the surgery group and the chemotherapy group. Patients with small tumor load and good general condition who were often included in the surgery group may gain better survival as bias in case selection may lead to differences in eventual survival. Hence, prospective randomized controlled studies are imperative.

Thus, we started to conduct this prospective, randomized, controlled phase II clinical study in late 2016 to determine the value of surgical resection of colorectal cancer in patients with unresectable metastases.

Reference

［1］Jemal A, Bray F, Center MM, et al. Global cancer statistics［J］.CA Cancer J Clin,2011,61(2):69-90.

［2］Xu J, Qin X, Wang J, et al. Chinese guidelines for the diagnosisand comprehensive treatment of hepatic metastasis of colorectalcancer[J].Jcancer Res Clin Oncol,2011,137(9):1379-1396.

［3］Abdalla EK. Commentary: Radiofrequency ablation for colorectal liver metastases: do not blame the biology when it is the technology［J］. Am J Surg, 2009, 197(6): 737-739.

［4］ De Jong MC, Pulitano C, Ribero D, et a1. Rates and patterns of recurrence following curative intent surgery for colorectal liver metastasis: an international multi-institutional analysis of 1669 patients［J］. Ann surg, 2009, 250(3): 440-448.

［5］ Poultsides GA, Servais EL, Saltz LB, et al. Outcome of primary tumor in patients with synchronous stage IV colorectal cancer receiving combination chemotherapy without surgery as initial treatment［J］. J Clin Oncol, 2009, 27(20): 3379-3384.

［6］Nitzkorski JR, Farma JM, Watson JC,et al. Outcome and natural history of patients with stage IV colorectal cancer receiving chemotherapy without primary tumor resection .［J］Ann Surg Oncol. 2012 Feb;19(2):379-83. doi: 10.1245/s10434-011-2028-1. Epub 2011

Aug 23.

［7］Tebbutt NC, Norman AR, Cunningham D,et al. Intestinal complications after chemotherapy for patients with unresected primary colorectal cancer and synchronous metastases. ［J］Gut. 2003 Apr;52(4):568-73

［8］Seo GJ, Park JW, Yoo SB, Kim SY,et al. Intestinal complications after palliative treatment for asymptomatic patients with unresectable stage IV colorectal cancer. ［J］J Surg Oncol. 2010 Jul 1;102(1):94-9. doi: 10.1002/jso.21577.

［9］Boselli C, Renzi C, Gemini A, et al. Surgery in asymptomatic patients with colorectal cancer and unresectable liver metastases: the authors' experience［J/OL］. Onco Targets Ther, 2013, 6:267-272. Doi: 10.2147/OTT.S39448.

［10］Sabine Venderbosch, MSc, Johannes H. de Wilt,et al. Prognostic Value of Resection of Primary Tumor in Patients with Stage IV Colorectal Cancer: Retrospective Analysis of Two Randomized Studies and a Review of the Literature. ［J］Ann Surg Oncol (2011) 18:3252–3260

［11］Aslam MI, Kelkar A, Sharpe D, et al. Ten years experience of managing the primary tumours in patients with stage IV colorectal cancers［J］. Int J Surg, 2010,8(4):305-313.

［12］Stillwell AP, Buettner PG, Ho YH. Meta-analysis of survival of patients with stage IV colorectal cancer managed with surgical resection versus chemotherapy alone［J］. World J Surg, 2010,34(4): 797-807.

2. Study objectives

Primary objectives:

This study is mainly aimed at comparing the time to failure of strategy(TTF) in patients treated with XELOX or mFOLFOX6 combined with or without surgical resection of the primary lesion, calculated from randomization to secondary progression in patients receive re-introduce of the original induction chemotherapy regimen after maintenance therapy, or to the first progression in patients without re-introduce of the original regimen, or to the time of primary lesion failure including colon perforation, bleeding, or ileus which needs local intervention.

Secondary objectives:

(1)Overall survival (OS);

(2) PFS: progression-free survival, i.e., the time from randomization to first progression;

(3) Chemotherapy tolerance;

(4) Quality of life;

(5) Objective response rate (ORR): ORR of induction therapy and re-induction therapy;

(6) Surgical complications: anastomotic leakage, hemorrhage, stenosis, etc.;

(7) Proportion of surgical interventions for primary lesion in the control group;

(8) Detection of relevant biomarkers and prognostic analysis.

3. Overview of study design

This trial is an open-label, prospective, randomized, controlled phase II clinical study, which is designed to compare the time to strategy failure (TTF) in patients treated with chemotherapy combined with or without surgical resection of the primary lesion. In this study, 130 patients with advanced colon cancer are planned to be included in our center.

Colon cancer Patients with unresectable metastases at enrollment will be randomly allocated to either the trial group (Group A) or the control group (Group B) and stratified by tumor response and the number of organ metastases, after receiving induction chemotherapy with 6 cycles of the mFOLFOX6 regimen or 4 cycles of the XELOX regimen, excluding progressors, those who can be radically resected, or those whose primary lesion remained palliatively unresectable (if patients have received chemotherapy with the XELOX or mFOLFOX6 regimen prior to enrollment, the original regimen chemotherapy will be continued until a total of 4 cycles of XELOX or 6 cycles of mFOLFOX6 has completed, then evaluated and randomized). Patients in the trial group receive surgical resection of the primary lesion and continue the original regimen chemotherapy after surgery (3-4 weeks) with 6 cycles of the mFOLFOX6 or 4 cycles of the XELOX regimen. Patients in the control group continue the original regimen chemotherapy with 6 cycles of the mFOLFOX6 or 4 cycles of the XELOX. Patients who do not progress during consolidation chemotherapy enter the Xeloda maintenance chemotherapy phase. During maintenance therapy, if progression occurs within 3 months after discontinuation of oxaliplatin, the second-line regimen will be performed; if progression occurs beyond 3 months after discontinuation of oxaliplatin and toxicity has recovered to grade I, the original regimen will be applied again, and the second-line regimen will be carried out unless progression occurs again. It is expected that 130 patients will be randomized after enrollment with 5 cases per month, and the enrollment will be completed in 2 years and ended in 3 years.

4. Study population

This is a prospective, randomized, controlled phase II clinical study, in which patients are randomized to the surgical treatment group or to the consolidation chemotherapy group to continue the original regimen after 2 evaluations (after induction chemotherapy with 6 cycles of the mFOLFOX6 regimen or 4 cycles of the XELOX regimen), excluding those who can be radically resected, or those who have progressed, or those whose primary lesion remain palliatively unresectable.

4.1 Inclusion criteria

Subjects are required to meet all of the following criteria to be enrolled in the study:

- Aged 18-80 years and of any gender;
- Eastern Cooperative Oncology Group (ECOG) physical status score (PS) 0 to 1, with an expected survival of more than 6 months;
- Histologically or cytopathologically confirmed colon adenocarcinoma;
- Presence of measurable or evaluable lesions;
- The primary lesion coexists with metastases, and distant metastases are unresectable;
- Within 7 days (including 7 days) before screening, subjects are required to meet the laboratory test data requirements: neutrophil count ≥ 1.5 × 10^9^/L, platelet count ≥ 90 × 10^9^/L, hemoglobin ≥ 90 g/L, serum total bilirubin ≤ 1.0 × upper limit of normal (ULN); ALT and AST ≤ 2.5 × ULN (≤ 5 × ULN for patients with liver metastases); serum creatinine ≤ 1.5 × ULN.
- Subjects have never received chemotherapy for metastatic disease;
- Subjects (or their legal representative/guardian) are required to sign an informed consent form indicating that they understand the study objectives, the procedures necessary for the study, and are willing to participate in the study.

4.2 Exclusion criteria

Anyone with one or more of the following conditions should not be included in this study:

- Presence of intestinal perforation, hemorrhage and complete obstruction requiring surgical intervention;
- Multiple primary colon cancers;
- Malignant peritoneal effusion;
- Uncontrollable pleural effusion;
- History of other cancer in the past five years, except for cured cervical cancer or cutaneous basal cell carcinoma;
- With brain or meningeal metastases;
- Pregnant or breast-feeding women; or those who are fertile but have not taken adequate contraceptive measures;
- Alcohol or drug addiction;
- With significant organ failure or other serious diseases, including symptomatic coronary artery disease, cardiovascular disease, or myocardial infarction within 12 months before enrollment; history of severe neurological or psychiatric illness; severe infection; active disseminated intravascular coagulation;
- patients had indications for the application of targeted therapy (bevacizumab or cetuximab), and could economically afford them..

4.3 Withdrawal criteria

- Pregnancy events occur in subjects during the course of the study.
- Disease progression is evaluated by imaging techniques;
- Subjects withdraw from the study;
- Subjects who delay treatment for more than two weeks for any reason during induction therapy (i.e., delay in all drugs in the dosing regimen);
- Other situations that the investigator considers necessary to withdraw from the study.

4.4 Elimination criteria

- Patients who do not enroll and complete the screening treatment according to the above case selection criteria and study protocol;
- Patients who have poor compliance and fail to follow the study protocol or switch to other chemical drugs during the study;
- Patients who enroll incorrectly owing to missing key parameters and incomplete study data.

5.Dosage and administration methods

Induction chemotherapy:

**Dosage and administration methods**

| XELOX regimen (4 cycles)： |
| --- |
| Oxaliplatin 130mg/m² ivgtt 2 hours, D1  Capecitabine 1000mg/m² P.O. bid，D1-D14  The regimen is repeated every 3 weeks and evaluated every 6 weeks. |

| mFOLFOX6 regimen (6 cycles)： |
| --- |
| Oxaliplatin 85mg/m² ivgtt 2 hours, D1,  CF 400mg/m² ivgtt 1 hour**，**D1,  5-FU 400mg/m² iv, D1，  5-FU 2.4g/m² CIV46h，  The regimen is repeated every 2 weeks and evaluated every 6 weeks. |

Consolidation chemotherapy:

The trial group: subjects continue receiving the original chemotherapy after surgery with 6 cycles of the mFOLFOX6 regimen or 4 cycles of the XELOX regimen.

The control group: subjects continue the original chemotherapy with 6 cycles of the mFOLFOX6 regimen or 4 cycles of the XELOX regimen.

Evaluation is performed every 6 weeks.

Maintenance chemotherapy: non-progressors enter Xeloda maintenance chemotherapy phase. Evaluation is performed every 2 months.

Follow-up treatment: during maintenance therapy, if progression occurs within 3 months after discontinuation of oxaliplatin, the second-line regimen will be performed; if progression occurs beyond 3 months after discontinuation of oxaliplatin and toxicity has recovered to grade I, the original regimen will be applied again, and the second-line regimen will be carried out unless progression occurs again. Imaging evaluation is performed every 2 months.

1. Study procedures

Colon cancer Patients with unresectable metastases at enrollment will be randomly allocated to either the trial group (Group A) or the control group (Group B) and stratified by tumor response and the number of organ metastases, after receiving induction chemotherapy with 6 cycles of the mFOLFOX6 regimen or 4 cycles of the XELOX regimen, excluding progressors, those who can be radically resected, or those whose primary lesion remained palliatively unresectable (if patients have received chemotherapy with the XELOX or mFOLFOX6 regimen prior to enrollment, the original regimen chemotherapy will be continued until a total of 4 cycles of XELOX or 6 cycles of mFOLFOX6 has completed, then evaluated and randomized). Patients in the trial group receive surgical resection of the primary lesion and continue the original regimen chemotherapy after surgery (3-4 weeks) with 6 cycles of the mFOLFOX6 or 4 cycles of the XELOX regimen. Patients in the control group continue the original regimen chemotherapy with 6 cycles of the mFOLFOX6 or 4 cycles of the XELOX. Patients who do not progress during consolidation chemotherapy enter the Xeloda maintenance chemotherapy phase. During maintenance therapy, if progression occurs within 3 months after discontinuation of oxaliplatin, the second-line regimen will be performed; if progression occurs beyond 3 months after discontinuation of oxaliplatin and toxicity has recovered to grade I, the original regimen will be applied again, and the second-line regimen will be carried out unless progression occurs again.

Stratified randomization: Patients will be randomly allocated and stratified by the number of metastatic sites (1 or more) and tumor response (PR or SD) after 2 evaluations and excluding those who can be radically resected, or those who have progressed including primary lesion failure(colon perforation, bleeding, or ileus which needs local intervention), or those whose primary lesion remained palliatively unresectable.

Group A (trial group): surgical treatment
 Group B (control group): continue chemotherapy

**Flow diagram:**

**
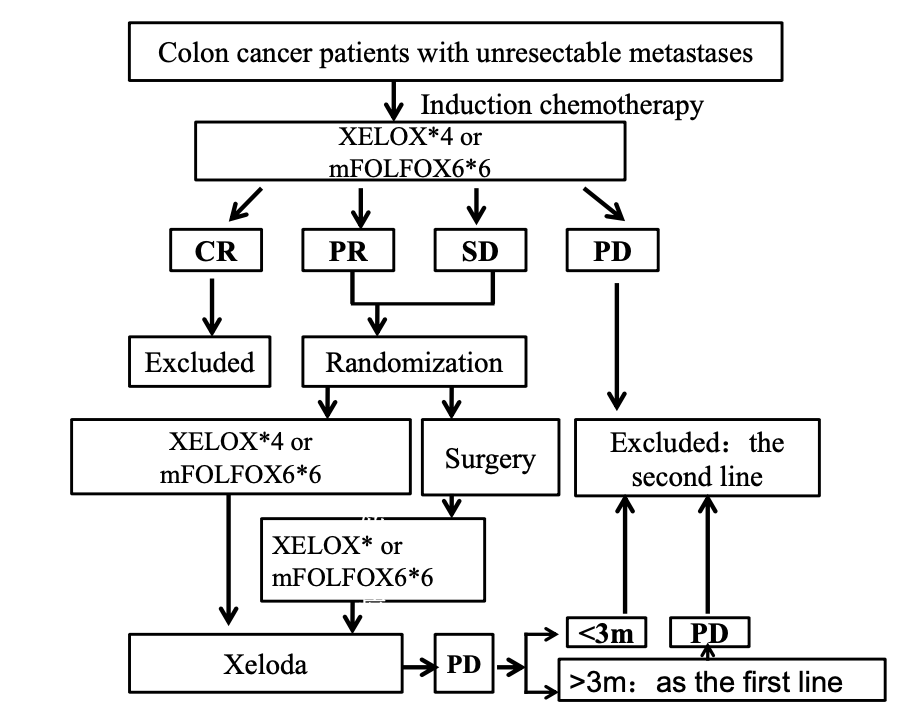
**

1. Compliance

The investigator should emphasize to the subject or his/her guardian the necessity of receiving accurate and timely medical treatment, surgical treatment or chemotherapeutic treatment throughout the study.

**8.**Concomitant therapy and concomitant medication

Concomitant disease: any disease already present at the start of the trial (i.e., the first visit).

Concomitant medication: any medication other than the investigational medication administered during the trial.

All concomitant diseases and concomitant medications should be recorded in detail when the subject enters the trial (i.e., the first visit). At each visit, any changes in concomitant medication should be recorded in detail. If the change has an impact on the subject's eligibility for continuation of the trial, the sponsor should be notified.

The following information should be collected for each concomitant medication at least: generic name, dosage, start date, stop date, continue administration, and indications.

9.Study evaluations

9.1 Study Steps

**Screening period/Baseline (-2W-0):**

The investigator should explain to each subject the risks and requirements of this clinical study as well as the complete information of the chemotherapy regimen treatment and its role in the trial. The investigator should also explain to them the objectives and requirements of this clinical study, and encourage them to pose questions about this trial. All subjects who volunteer to participate in this trial are required to read and sign an informed consent form to participate in this clinical study before all relevant trial procedures are performed. After obtaining the subject's signed informed consent form, the investigator will evaluate whether the subject is eligible for the trial based on the inclusion criteria and exclusion criteria. If the investigator determines that the subject is eligible for the trial, the following evaluations and procedures should be completed.

9.2 Efficacy Evaluation

Patients who achieve SD or PR after 2 evaluations (6 cycles of the mFOLFOX6 or 4 cycles of the XELOX), and with no bleeding, or perforation, or obstruction; no malignant peritoneal effusion; patients whose metastases cannot be completely resected after re-evaluation will be randomized into the chemotherapy group or the surgical treatment group.

Objective response rate, progression-free survival and overall survival are applied to efficacy evaluation. This study protocol will continue until disease progression or intolerable adverse reactions occur, and the time to disease progression and overall survival will be evaluated during each visit and follow-up period. RECIST 1.1 criteria will be adopted in efficacy evaluation.

9.3 Safety Evaluation

NCI – CTCAE version 4.0 will be adopted to evaluate the clinical safety of the treatment in the study. The occurrence of adverse events in subjects should be evaluated at each clinical visit.

Adverse events

Subjects (or their legal representatives when appropriate ) should report any adverse event that occurred during the course of the trial.

The investigator should collect, follow up and report adverse events according to the specified procedures. See Section 13 for further details on adverse events. Any clinical abnormalities that are ongoing at the end of the study should be followed up until response or clinical stability is reached.

10.Toxicities and dose adjustments

(1) Close monitoring of subjects’ toxicity should be performed and the dose of oxaliplatin and xeloda should be adjusted based on the most severe toxicity that occurred in the previous treatment cycle.

(2) A maximum of 2 weeks’ delay in treatment is allowed so as to make subjects recover from treatment-related toxicity. If the subject remains unrecovered after a 2-week delay, the subject should be discontinued from the treatment.

(3) Patients who require more than 2 dose adjustments should be discontinued from the trial.

(4) If the following hematological toxicities occur, Grade IV leukopenia or neutropenia, Grade-III febrile neutropenia and Grade III or IV thrombocytopenia, a 25% dose reduction of oxaliplatin and xeloda should be performed in the next course of treatment. If the above toxicities still occur after the first dose reduction, the dose of oxaliplatin and xeloda should be reduced by another 25%.

(5) If Grade III or IV non-hematological toxicities (except for alopecia, neurotoxicity and diarrhea) occurs and the investigator considers that the toxicity is just related to one drug, then no dose adjustment of other drugs is required and the dose of the related drug is reduced by 25% for the next course of treatment. If the above toxicities still occur after the first dose reduction, the relevant drug can be reduced by 25% for the second time.

(6) If special situations not mentioned above occur, then the dose adjustment is at the discretion of the investigator.

| NCI toxicity grade | Chemotherapeutic dose level for next cycle | At the beginning of the next cycle |
| --- | --- | --- |
| Neutropenia | | |
| I | Maintain the original dose level | If neutrophils are less than 1.5×10^9^/L at the beginning of a chemotherapy cycle, the drug should be suspended. |
| II | Maintain the original dose level |
| III | Maintain the original dose level |
| IV | Reduce by one dose level |
| Febrile neutropenia | Reduce by one dose level |
| Thrombocytopenia | | |
| I | Maintain the original dose level | If platelets are less than 7.5×10^9^/L at the beginning of a chemotherapy cycle, the drug should be suspended. |
| II | Maintain the original dose level |
| III | Reduce by one dose level |
| IV | Reduce by one dose level |
| Non-hematological toxicities (except for alopecia, neurotoxicity and diarrhea) | | |
| I | Maintain the original dose level | If non-hematological toxicity is greater than or equal to Grade II at the beginning of a chemotherapy cycle, the drug should be suspended. |
| II | Maintain the original dose level |
| III | Reduce by one dose level |
| IV | Reduce by one dose level |

1. If Grade III neurotoxicity occurs, adjust the dose of oxaliplatin; if Grade IV neurotoxicity occurs, discontinue oxaliplatin and treat with xeloda only or CF/5-FU.
2. If Grade III hand-foot syndrome occurs, change XELOX regimen to mFOLFOX6 regimen (3 cycles of mFOLFOX6 regimen is equivalent to 2 cycles of XELOX regimen chemotherapy).

11.Concomitant therapy

Treatment of vomiting

Patients can be given prophylactic antiemetic therapy and appropriate fluid replacement therapy.

Treatment of diarrhea

Patients can be given antidiarrheal and fluid replacement supportive therapy.

Treatment of allergic reactions

If allergy occurs during oxaliplatin dripping, then symptomatic treatment should be immediately given according to conventional treatment experience.

Treatment of colony stimulating factor

Granulocyte-colony stimulating factor (G-CSF) is not used prophylactically, but can be used when Grade II myelosuppression occurs.

Treatment of febrile neutropenia

Patients who have developed febrile neutropenia with or without diarrhea should be hospitalized for symptomatic treatment such as antibiotics and leukocyte elevating therapy. Electrolytes should also be monitored.

Other underlying diseases should be treated according to the original treatment if there are no liver enzymes or drug interactions. If liver enzymes or drug interactions are present or suspected, then the treatment will be discussed by the investigators.

Drugs prohibited or used with caution during the study

Modern traditional Chinese medicine preparations and immunomodulators (such as thymosin, interferon, interleukin-2 and lentinan, etc.) approved by SFDA against colorectal cancer are prohibited in this protocol.

12.Completion/premature withdrawal of subjects

Omitted

13.Statistical analysis

1. Analysis of data sets

All eﬃcacy analyses will be made mainly in the intention-to-treat (ITT) population (all randomised patients, irrespective of whether they received study medications)，and in Per Protocol Set(PPS) as supplement. PPS is defined as subjects with no serious violations of the study protocol,

.AE will be evalued in the safety set (SS): Subjects who have received at least one chemotherapy and at least one safety evaluation after randomization will be included in the SS of the study.

1. Statistical methods

Descriptive statistics will be adopted to analyze all the data, including demographic data, baseline, various efficacy evaluation parameters and all safety data. The measurement data will be analyzed by describing the mean, standard deviation, number of valid cases, median, maximum and minimum, and calculating 95% confidence interval of the mean. The enumeration data will be analyzed by describing the frequency and percentage and calculating 95% confidence interval of the percentage. Whether the parameters meet normality will be evaluated by the efficacy before and after treatment. Intra-group comparisons of measurement data will be performed using paired t-test or Wilcoxon rank sum test for paired data. Rank data will be analyzed using the Wilcoxon rank sum test, and enumeration data will be compared using the χ^2^ test or Fisher's exact probability test. Unless otherwise specified, all hypothesis tests used in this study are two-sided tests with 0.05 as the test level.

1. Calculation of sample size

A number of previous phase III clinical studies have shown that the PFS of advanced colorectal cancer is about 8 months. This clinical study is a superiority test. As patients received a total of 3-month induction chemotherapy in this trial, so after randomization, the median TTF (mTTF) might be about 5 months in chemotherapy alone group (group B). and it might be longer in surgical group (group A) as resection of primary tumor might reduce the rate of primary lesion failure. The mTTF was assumed to be 5 months in group B and 9 months in group A, which get the clinical valuable absolute benefit of 4 months prolong, with a corresponding hazard ratio (HR) of 0.556. Based on HR = 0.8, α = 0.05 and 20% dropout rate, a total of 130 patients (number of cases after randomization) are required in this study.

1. Analysis of primary efficacy parameters

Descriptive statistical analysis will be performed for efficacy endpoints.

1. Analysis of secondary efficacy parameters

Descriptive statistical analysis of rating scales and hypothesis tests for improvement from baseline will be performed at the endpoint.

1. Safety parameters

Subjects who have received at least one chemotherapy and at least one safety evaluation will be included in the safety analysis. Descriptive statistical analysis will be adopted for safety analysis.

14.Reporting of adverse events

Omitted

15. Ethics
Omitted
